# Examining the Public’s Most Frequently Asked Questions Regarding COVID-19 Vaccines Using Search Engine Analytics in the United States: Observational Study

**DOI:** 10.2196/28740

**Published:** 2021-08-04

**Authors:** Nicholas B Sajjadi, Samuel Shepard, Ryan Ottwell, Kelly Murray, Justin Chronister, Micah Hartwell, Matt Vassar

**Affiliations:** 1 Office of Medical Student Research College of Osteopathic Medicine Oklahoma State University Center for Health Sciences Tulsa, OK United States; 2 Department of Internal Medicine University of Oklahoma School of Community Medicine Tulsa, OK United States; 3 Department of Dermatology St. Joseph Mercy Hospital Ann Arbor, MI United States; 4 Department of Emergency Medicine College of Osteopathic Medicine Oklahoma State University Center for Health Sciences Tulsa, OK United States; 5 Department of Internal Medicine College of Osteopathic Medicine Oklahoma State University Center for Health Sciences Tulsa, OK United States; 6 Department of Psychiatry and Behavioral Sciences College of Osteopathic Medicine Oklahoma State University Center for Health Sciences Tulsa, OK United States

**Keywords:** content, COVID-19, frequently asked questions, internet, machine learning, natural language processing, quality, question, SARS-CoV-2, search analytics, search engine, transparency, vaccine hesitancy, vaccine, web-based health information

## Abstract

**Background:**

The emergency authorization of COVID-19 vaccines has offered the first means of long-term protection against COVID-19–related illness since the pandemic began. It is important for health care professionals to understand commonly held COVID-19 vaccine concerns and to be equipped with quality information that can be used to assist in medical decision-making.

**Objective:**

Using Google’s RankBrain machine learning algorithm, we sought to characterize the content of the most frequently asked questions (FAQs) about COVID-19 vaccines evidenced by internet searches. Secondarily, we sought to examine the information transparency and quality of sources used by Google to answer FAQs on COVID-19 vaccines.

**Methods:**

We searched COVID-19 vaccine terms on Google and used the “People also ask” box to obtain FAQs generated by Google’s machine learning algorithms. FAQs are assigned an “answer” source by Google. We extracted FAQs and answer sources related to COVID-19 vaccines. We used the Rothwell Classification of Questions to categorize questions on the basis of content. We classified answer sources as either academic, commercial, government, media outlet, or medical practice. We used the Journal of the American Medical Association’s (JAMA’s) benchmark criteria to assess information transparency and Brief DISCERN to assess information quality for answer sources. FAQ and answer source type frequencies were calculated. Chi-square tests were used to determine associations between information transparency by source type. One-way analysis of variance was used to assess differences in mean Brief DISCERN scores by source type.

**Results:**

Our search yielded 28 unique FAQs about COVID-19 vaccines. Most COVID-19 vaccine–related FAQs were seeking factual information (22/28, 78.6%), specifically about safety and efficacy (9/22, 40.9%). The most common source type was media outlets (12/28, 42.9%), followed by government sources (11/28, 39.3%). Nineteen sources met 3 or more JAMA benchmark criteria with government sources as the majority (10/19, 52.6%). JAMA benchmark criteria performance did not significantly differ among source types (*χ*^2^_4_=7.40; *P*=.12). One-way analysis of variance revealed a significant difference in mean Brief DISCERN scores by source type (*F*_4,23_=10.27; *P*<.001).

**Conclusions:**

The most frequently asked COVID-19 vaccine–related questions pertained to vaccine safety and efficacy. We found that government sources provided the most transparent and highest-quality web-based COVID-19 vaccine–related information. Recognizing common questions and concerns about COVID-19 vaccines may assist in improving vaccination efforts.

## Introduction

As of August 01, 2021, COVID-19 has affected over 198 million people and has been responsible for over 4.2 million deaths worldwide [1,2]. In response to the pandemic, the US Food and Drug Administration issued emergency use authorizations for 2 COVID-19 vaccines in late 2020, 1 manufactured by Pfizer-BioNTech and the second by Moderna [3,4]. Overcoming logistical barriers will be crucial for enabling successful vaccine campaigns. Additionally, addressing the public’s perception of COVID-19 vaccines and the quality of available information is vital for promoting positive public reception and reducing vaccine hesitancy. Vaccine hesitancy, which refers to reluctance or refusal to receive vaccines, is complex and is determined by numerous factors such as trust in vaccine safety and efficacy, perceived risk of receiving or refusing a vaccine, and accessibility to and affordability of vaccines [5]. Hesitancy toward COVID-19 vaccines may hinder successful vaccination efforts.

The pace of vaccine development, misinformation, and overall growth in vaccine hesitancy are factors potentially contributing to COVID-19 vaccine refusal [5,6]>. Identifying factors associated with COVID-19 vaccine refusal may assist in developing strategies to reduce vaccine hesitancy. To identify demographic factors associated with COVID-19 vaccine acceptance, Lazarus et al [7] surveyed individuals in 19 countries and reported that individuals who reported a high degree of trust in the government were more likely to report vaccine acceptance than those with low trust. In the United States, a survey study by the US Census Bureau showed that 49% of respondents were reluctant to receive a COVID-19 vaccine. Of those reluctant to receive COVID-19 vaccines, the most common reason for reluctance was concern for side effects. The second most common reason was planning to wait and see if the vaccines were safe [8]. A US survey conducted early in the pandemic sought to predict COVID-19 vaccine acceptance in the United States and found that several vulnerable populations reported low willingness [9]. The growing prevalence of vaccine hesitancy highlights the importance of clinician preparedness to address patients’ concerns as access to COVID-19 vaccines grows. Health care professionals should serve as reliable sources of vaccine information, instilling confidence in patients and potentially enhancing vaccine acceptance [10], especially for COVID-19 vaccines [11].

Apart from consulting health care professionals, individuals frequently use the internet when seeking health care information; some use the internet as their primary source for health information [12]. In the United States, 61% of adults have searched the internet for medical information [13]. Searching the internet for medical information simultaneously presents benefits and challenges regarding patient-provider interactions [14]. The increasingly common practice of using the internet to obtain health care information makes it possible to study commonly held medical concerns by examining searching patterns and behaviors. Previous studies have documented the prevalence of COVID-19 vaccine hesitancy in the United States [8,15] and globally [7], but none of these studies explored the content of COVID-19 vaccine concerns evidenced by internet searching. Moreover, the quality of COVID-19 vaccine information resulting from internet searching has yet to be investigated. Thus, the primary objective of this study was to use Google’s RankBrain machine learning algorithm to characterize the content of the most frequently asked questions (FAQs) about COVID-19 vaccines in the United States. Secondarily, we sought to grade the transparency and quality of suggested information regarding COVID-19 vaccines. We aim to equip health care professionals and researchers with information about the common concerns regarding COVID-19 vaccines, possibly supporting more successful vaccination efforts. We hypothesize that most COVID-19 vaccine–related FAQs in the United States will pertain to safety and efficacy, as survey studies have indicated these concerns as the most important driver of COVID-19 vaccine hesitancy in the United States.

## Methods

### Background

We used Google to perform our search as it is the most frequently used search engine globally as of 2015 [16]. Moreover, Google’s search engine uses a powerful machine learning system called RankBrain [17] alongside the natural language processing technology known as Bidirectional Encoder Representations from Transformers [18] to detect patterns from large volumes of search queries. Google assesses the intent of a search query using rigorous language processing algorithms to sort through billions of indexed webpages and to suggest the ones most relevant to the search [19]. The resulting patterns and data are used to formulate lists of FAQs related to the original search contents. FAQs are found in boxes labeled “People also ask” or “Common questions.” Google assigns each FAQ a link to information that “answers” the question [20]. Google uses its webmaster guidelines to remove low-quality spam websites from search results and prioritize high-quality sources using a system called PageRank [19]. Taken together, these FAQs represent millions of common inquiries regarding medical information. Linked answers to each FAQ reveal which information sources individuals are likely to encounter when searching Google for medical information. Our methodology was adapted from a study by Shen et al [21], who used Google FAQs to reliably reveal common concerns about orthopedic procedures and to assess the transparency of the suggested information.

### Systematic Search

On January 23, 2021, using a newly installed web browser to minimize personalized advertisement algorithms, we separately searched Google [22] for the following three terms: “covid 19 vaccine,” “pfizer covid vaccine,” and “moderna covid vaccine.” We selected these terms to capture the most likely general inquiries concerning the only 2 COVID-19 vaccines available at the time of our search. For each inquiry, we refreshed the list of FAQs found in the “Common questions” or “People also ask” box generated by Google. By expanding the tab on a FAQ, additional FAQs appear. We repeated this process until reaching a minimum of 150 FAQs for each search, as studies using similar methodology have recommended using 50-150 sources [21]. We used the high end of the recommended number of sources (150) for two reasons: to increase the likelihood of encountering an FAQ that would be pertinent to the current study and to reflect the precedent set in the literature. Since query results are tailored to the user’s location, search history, and search settings, we used clean browsers to minimize any influence of history and settings while allowing results to reflect queries from the United States [19].

### Data Extraction

Of the resultant FAQs, we extracted only those directly pertaining to or mentioning COVID-19 vaccines along with their answer links. In a masked duplicated fashion, investigators NS and SS extracted these data using a Google Form on January 23, 2021. FAQ data extraction was completed on January 23, 2021. After extraction, any duplicate FAQs from the individual searches were removed, followed by the removal of any duplicate FAQs among the 3 searches. After the screening and reduction process, our searches resulted in a compilation of unique FAQs regarding COVID-19 vaccines.

### Question Classification and Answer Source Type

Applying methodology adapted from previous studies [16,21], we first used the Rothwell Classification of Questions [23] to categorize FAQs under three broad categories: fact, policy, and value. Fact questions were further subclassified into four groups: safety and efficacy, vaccine administration schedule, cost, and technical details. Policy questions were subclassified into two groups: indications and complications. Value questions were subclassified into two groups: evaluation of credibility and appraisal of risk or benefit. Next, we categorized answer sources as either commercial, academic, medical practice, government, or media outlet according to previously established classification schemes [21,24]. Table 1 shows the *Question Classification* and *Answer Source Type* definitions. For each answer source, we extracted the country of origin.

**Table 1:** The Rothwell Classification of Questions, Question Classification by Topic, and Answer Source Type.

Rothwell classification				Description
Fact				Asks objective, factual information regarding COVID-19 vaccines (ie, “How long does it take the vaccine to work?”)
Policy				Asks information on a specific course of action under given circumstances related to COVID-19 (ie, should people on immunosuppressants get the vaccine?)
Value				Asks to conceptually evaluate COVID-19 vaccines (ie, “Will the COVID-19 vaccine work better than masks?”)
**Question subclassification by topic**				
	**Fact**			
		Safety and efficacy	Questions about vaccine safety including side effects and how well the vaccine works	
		Vaccine administration schedule	Specific questions about the vaccine schedule, number of shots, and vaccine distribution	
		Cost	Cost of the vaccine, whether it is free, or who is paying for it	
		Technical Details	Mechanism by which the vaccine works, including specific questions about immunologic responses	
	**Policy**			
		Indications	Who should or should not receive a COVID-19 vaccine	
		Complications	Questions about specific complications after being vaccinated	
	**Value**			
		Evaluation of Credibility	Seeking authoritative approval from a trustworthy source; seeking ethos	
		Appraisal of Risk or Benefit	Necessity of preventive measures after vaccination (ie, “Is getting vaccinated worth it?”)	
**Answer source type**				
	Commercial		Organization that publishes medical information that is not otherwise associated with an academic institution, government agency, health care system, or nonmedical news outlet such as WebMD and Healthline	
	Academic		Institution with clear academic affiliations, as evidenced by information on the website that did not better meet criteria for another classification or website ending in “*.edu*,” such as Mayo Clinic and Harvard University	
	Medical practice		Affiliation with a health care system or individual health care professional who did not explicitly state a commercial, academic, or government affiliation, such as private practice and a hospital system	
	Government		Websites hosted by government organizations or sources from websites ending in “.gov,” such as the Centers for Disease Control and the US Food and Drug Administration	
	Media outlet		Nonmedical organizations or social media pages claiming to publish news-related stories for the purpose of information-sharing in the form of interviews, blog posts, or articles, such as the National Public Radio, Wall Street Journal, and USA Today	

### Information Transparency and Quality

The Journal of the American Medical Association’s (JAMA’s) benchmark criteria [25] was then used to assess information transparency for each answer source. JAMA benchmark criteria have been used to effectively screen web-based information for fundamental aspects of information transparency [21,26-28]. JAMA benchmark criteria were also used to characterize web-based misinformation regarding COVID-19 in early 2020 [29]. Sources meeting 3 more criteria are considered to have high transparency, while sources meeting less than 3 criteria have poor transparency. Table 2 lists the JAMA benchmark criteria definitions.

**Table 2:** Journal of the American Medical Association’s benchmark criteria.

Criteria	Description
Authorship	Clearly identifiable author and contributors with affiliations and relevant credentials present.
Attribution	References and sources clearly listed with any copyright information disclosed.
Currency	Clearly identifiable posting date of any content as well as the date of any revisions.
Disclosure	Website ownership clearly disclosed along with any sponsorship, advertising, underwriting, and financial support.

The information quality was assessed using the Brief DISCERN information quality assessment tool. DISCERN is a series of questions originally developed by Charnock et al [30] as a means for patients and providers to quickly and reliably ascertain the quality of written health care information regarding medical treatments. The DISCERN quality assessment tool has been used to assess the quality of internet sources in a variety of medical fields [31-33]. Khazaal et al [34] developed an abbreviated 6-item version (Brief DISCERN) with comparable reliability and validity, which preserves the advantages of the original tool while affording a potentially more user-friendly format. Thus, we used the Brief DISCERN quality assessment tool, which has been previously used [35,36]. Sources are scored from 1 to 5 based on the criteria listed in Table 3.

Authors NS and SS applied the JAMA benchmark criteria and the Brief DISCERN tool in a masked duplicate fashion, and author MH resolved any discrepancies. This protocol was submitted to the institutional review board of Oklahoma State University Center for Health Sciences and was determined to be non–Human Subjects Research.

**Table 3:** Brief DISCERN questions and scoring.

Questions	Score		
	Low (1) “No”	Moderate (2-4) “Partially”	High (5) “Yes”
Is it clear what sources of information were used to compile the publication (other than the author or producer)?	No sources of evidence for the information are mentioned	The sources are clear to some extent and are referenced in the text *or* in a bibliography	The sources are very clear and are referenced in text *and* in a bibliography
Is it clear when the information used or reported in the publication was produced?	No dates have been given	Only the date of the publication itself is clear, or dates for some of but not all acknowledged sources are given	Dates for all acknowledged sources are clear
Does it describe how each treatment works?	None of the descriptions about treatments include details of how it works	Descriptions of some but not all treatments are given *or* the details provided are unclear or incomplete	The description of treatment includes details of how it works
Does it describe the benefits of each treatment?	No benefits are described	A benefit is described for some but not all treatments	A benefit is described for each treatment
Does it describe the risk of each treatment?	No risks are described for any of the treatments.	A risk is described for some but not all treatments.	A risk is described for each treatment.
Does it describe how the treatment choices affect overall quality of life?	There is no reference to overall quality of life in relation to treatment choices.	The publication includes a reference to overall quality of life in relation to treatment choices, but the information is unclear or incomplete.	The publication includes a clear reference to overall quality of life in relation to any of the treatment choices mentioned.

### Analyses

Frequencies and percentages were reported for each FAQ’s classification. Chi-square tests were used to determine associations between JAMA benchmark criteria by source type. One-way analysis of variance was used to determine whether the mean Brief DISCERN score differed by source type. Post hoc comparisons, performed using *t* tests with Bonferroni correction, were used to identify mean differences between source type categories. Interrater agreement for each assessment was determined using intraclass correlation coefficients.

## Results

A total of 467 FAQs were generated from all 3 searches: 161 from “covid 19 vaccine,” 155 from “moderna covid vaccine,” and 151 from “pfizer covid vaccine.” Of these, “covid 19 vaccine” yielded 5 vaccine-related FAQs, “moderna covid vaccine” yielded 22, and “pfizer covid vaccine” yielded 14. After removing duplicates, our searches yielded a total of 28 unique FAQs regarding COVID-19 vaccines (Table 4).

**Table 4:** List of the 28 unique frequently asked questions regarding COVID-19 vaccines.

Frequently asked questions	Rothwell classification	Subclassification	Answer source	JAMA benchmark criteria (≥3)	Brief DISCERN score
Are both Covid vaccines 2 doses?	Fact	Vaccine administration schedule	Commercial	No	15
Are you immune to Covid after vaccine?	Fact	Safety and efficacy	Media outlet	No	21
Can I get COVID-19 right after being vaccinated?	Fact	Technical details	Government	Yes	29
Can the COVID-19 vaccine make you sick?	Fact	Safety and efficacy	Government	Yes	29
Can you still get Covid after first vaccine?	Fact	Technical details	Media outlet	No	18
Can you test positive for Covid after vaccine?	Fact	Technical details	Media outlet	No	9
Do COVID-19 vaccines require more than one shot?	Fact	Vaccine administration schedule	Government	Yes	29
Do you have to wait 90 days after Covid to get the vaccine?	Fact	Vaccine administration schedule	Media outlet	Yes	15
Do you have to wear mask after Covid vaccine?	Value	Risk/benefit appraisal	Media outlet	Yes	28
Does Covid vaccine Stop Spread?	Value	Risk/benefit appraisal	Media outlet	Yes	22
Has the Pfizer-BioNTech COVID-19 vaccine been authorized by the FDA?	Fact	Safety and efficacy	Government	Yes	30
How does the COVID-19 mRNA vaccine work?	Fact	Technical details	Government	No	25
How effective is the Pfizer COVID-19 vaccine?	Fact	Safety and efficacy	Media outlet	No	17
How long do you have to wait between Covid vaccines?	Fact	Vaccine administration schedule	Media outlet	No	16
How many shots of Moderna COVID-19 vaccine should I get?	Fact	Vaccine administration schedule	Government	Yes	29
Is it safe to take the COVID-19 vaccine?	Fact	Safety and efficacy	Government	Yes	29
Is the Moderna vaccine for COVID-19 approved by the FDA?	Fact	Safety and efficacy	Academic	Yes	30
Should you get the Covid vaccine if you were previously infected with Covid?	Policy	Indications	Media outlet	Yes	15
What are some common side effects of the COVID-19 vaccine?	Fact	Safety and efficacy	Government	Yes	29

### Question Classification

Using the Rothwell classification system, the majority of FAQs were seeking factual information (22/28;78.6%). Among these factual questions, the most common topic was safety and efficacy (9/22, 40.9%) followed by technical details (6/22, 27.3%), vaccine administration schedule (6/22, 27.3%), and cost (1/22, 4.5%) (Table 4).

### Answer Sources

The most common answer source type overall was media outlets (12/28, 42.9%), followed by government sources (11/28, 39.3%), commercial sources (3/28, 10.7%), academic sources (1/28, 3.55%), and medical practice (1/28, 3.55%). FAQs classified as technical details were most frequently answered by a media outlet (4/6, 66.7%). Of FAQs classified as fact, most were answered by government sources (11/22, 50%). Government sources also most commonly answered FAQs related to safety and efficacy (5/9, 55.6%), cost (1/1, 100%), and vaccine administration schedule (3/6, 50%) (Table 4). In total, 26 of 28 (92.8%) answer sources were from the United States, 1 was from the United Kingdom (3.6%), and 1 was from Australia (3.6%).

### Information Transparency

In total, 19 sources met 3 or more JAMA benchmark criteria, of which government sources were the majority (10/19, 52.6%), followed by media outlets (7/19, 36.8%), commercial sources (1/19, 5.3%), and academic sources (1/19, 5.3%). Among sources meeting less than 3 criteria, media outlets were the most common (5/9, 55.6%), followed by commercial sources (2/9, 22.2%), medical practice (1/9, 11.1%), and government sources (1/9, 11.1%). Approximately 92.7% (11/12) of government sources met 3 or more JAMA benchmark criteria, whereas 58.3% (7/12) of media outlets met 3 or more criteria. The overall JAMA Benchmark Criteria performance did not significantly differ among source types (*χ*^2^_4_=7.40; *P*=.12); however, we found significant associations between individual source’s performance on meeting JAMA benchmark criteria for authorship and the source type (*χ*^2^_4_*=18.03*, *P*<.001), with 11/28 (39.3%) media outlet sources meeting authorship criteria compared to 10/28 (35.7%) government sources not meeting the authorship criteria. We also found a similar but negative relationship with JAMA benchmark criteria’s disclosure criteria and source type (*χ*^2^_4_=15.36; *P*=.004) with 10/28 (35.7%) government sources meeting these criteria compared to 9/28 (32.1%) media outlets not meeting these criteria (Tables 5 and 6).

**Table 5:** Journal of the American Medical Association’s benchmark criteria and by source type.

Sources meeting 3 or more JAMA benchmark criteria		Source type, n (%)					Total	Chi-square (*df*)	*P* value
		Academic	Commercial	Government	Medical practice	Media outlet			
**Journal of the American Medical Association’s benchmark criteria**								7.40 (*4*)	.12
	3+	1 (3.6)	1 (3.6)	10 (35.7)	0 (0.0)	7 (25.0)	19 (67.9)		
	<3	0 (0.0)	2 (7.1)	1 (3.6)	1 (3.6)	5 (17.9)	9 (32.1)		
**Authorship**								18.03 (*4*)	.001
	No	1 (3.6)	2 (7.1)	10 (35.7)	0 (0.0)	1 (3.6)	14 (50.0)		
	Yes	0 (0.0)	1 (3.6)	1 (3.6)	1 (3.6)	11 (39.3)	14 (50.0)		
**Attribution**								7.21 (*4*)	.13
	No	0 (0.0)	2 (7.1)	1 (3.6)	1 (3.6)	4 (14.3)	8 (28.9)		
	Yes	1 (3.6)	1 (3.6)	10 (35.7)	0 (0.0)	8 (28.9)	20 (71.4)		
**Currency**								1.60 (*4*)	.81
	No	0 (0.0)	0 (0.0)	1 (3.6)	0 (0.0)	0 (0.0)	1 (3.6)		
	Yes	1 (3.6)	3 (10.7)	10 (35.7)	1 (3.6)	12 (42.9)	27 (96.4)		
**Disclosure**								15.36 (*4*)	.004
	No	0 (0.0)	3 (10.7)	1 (3.6)	1 (3.6)	9 (32.1)	14 (50.0)		
	Yes	1 (3.6)	0 (0.0)	10 (35.7)	0 (0.0)	3 (10.7)	14 (50.0)		

**Table 6:** Brief DISCERN scores by source type.

	Source type					Average (SD)	*F* value (*df*)	*P* value
	Academic	Commercial	Government	Medical practice	Media outlet			
Brief DISCERN score, mean (SD)	30.0 (0.0)	17 (2.6)	28.6 (1.4)	18.0 (0.0)	19.6 (5.6)	23.2 (6.2)	10.27 (*4, 23*)	<.001

### Information Quality

ANOVA revealed significant differences in mean Brief DISCERN scores by source type (*F*_4,23_=10.27; *P*<.001), suggesting important differences in quality among the different source types. Post hoc analysis with Bonferroni correction revealed significant differences in Brief DISCERN scores between government and commercial sources (*P*=.002) and between government sources and media outlets (*P*<.001). Mean (SD) values of Brief DISCERN scores by source are provided in Table 6. Interrater agreement for our analyses was high (interclass correlation=0.96; 95% CI 0.95-0.97).

## Discussion

### Principal Findings

Using Google and its search analytics, we were able to identify the most frequently asked questions regarding COVID-19 vaccines in the United States. Google generated these FAQs by using millions of search queries nationwide. Additionally, we evaluated the assigned “answer” source for each FAQ, assessing each source’s information transparency and quality. To our knowledge, this study is the first of its kind to evaluate the public’s most frequently asked questions concerning the COVID-19 vaccines in the United States using Google search analytics. Our study is also the first of its kind to identify common answer sources used to address COVID-19 vaccine–related concerns and to assess their transparency and quality. In the following discourse, we discuss the importance of knowing COVID-19 FAQs in the context of the current COVID-19 vaccination campaigns while also providing recommendations for improving the public’s confidence and willingness to be vaccinated.

### FAQs

The most popular COVID-19 vaccine–related questions sought factual information regarding safety and efficacy, indicating greater public concern regarding these topics. Consistent with our findings, survey studies found that safety and efficacy were among the most common COVID-19 vaccine concerns reported by the public and health care workers [37-40]. Additionally, studies have identified safety concerns as being one of the most common reasons for COVID-19 vaccine hesitancy [8,38-42]. In the United States, surveys indicate that 10% to 20% of adults and an estimated 8% of health care workers will refuse COVID-19 vaccines [8,37,39,43]. While the willingness to receive the COVID-19 vaccines has increased, the alarmingly high percentage of adults refusing vaccination creates a significant barrier to protecting our most vulnerable populations [43-45]. The potential cost of vaccine hesitancy and refusal in the United States is not exclusive to the COVID-19 pandemic. For example, an outbreak of measles virus, a pathogen for which vaccines effectively control outbreaks, occurred in Clark County, Washington, in 2019 [46]. Of 71 individuals involved, 61 (86%) were unvaccinated and 52 (73%) were children [46,47]. Moreover, vaccination rates in Clark County have been 10%-14% below the national average (88%) since 2013. The measles outbreak in 2019 was estimated to cost US $3.3 million to $3.5 million in labor, direct medical costs, and productivity losses [48]. It is likely that the cost of the Clark County measles outbreak could have been mitigated or reduced with adequate vaccination [47]. Thus, to prevent similar, but likely far worse, outcomes with COVID-19, effectively educating the public on the safety of COVID-19 vaccines is paramount for enhancing COVID-19 vaccine acceptance [49].

### Answer Sources

Overall, COVID-19 vaccine FAQs were most often answered by media outlets, followed by government sources. FAQs about safety and efficacy were answered more often by government sources, while media outlets frequently answered FAQs about technical details. The answer sources linked to each FAQ are found in “People also ask” or “Common concerns” boxes and are direct answers generated by Google [50]. These direct answers are supplied from Google’s “trusted entities” database and are based on relational topics and machine learning [50]. While “trusted entities” seems rather vague, it appears that Google considers direct answers to be “trusted” based on clarity, completeness, and the lack of excessive promotional jargon. With the public’s trust and willingness to accept the vaccine being a key element in a successful vaccination campaign [44,51-53], it may be more appropriate for direct answers addressing COVID-19 vaccine FAQs to be based on scientific integrity, objectivity, and transparency.

### Transparency and Quality of the Answer Source

The FAQs with direct answers from government sources were more likely to meet 3 or more JAMA benchmark criteria, indicating that government answers were more transparent. Additionally, government and academic sources were found to be of significantly higher quality. While media outlets are unquestionably an important source of health information to the public, these findings suggest that government sources may be better for addressing the public’s COVID-19 vaccine concerns. Although media outlets had moderate transparency and quality, there are notable reasons to use more reliable and objective sources. Generally, COVID-19 misinformation is rampant and the public opinion can be easily manipulated [29,45]. Indeed, media outlets are a frequent source of COVID-19 misinformation, and false claims are amplified by widespread news coverage [29,54]. For example, news stories early in the pandemic touting hydroxychloroquine as a “cure” perpetuated this misinformation in the absence of evidence [55]. More recently and more specifically related to the COVID-19 vaccines, rumors that COVID-19 vaccines cause infertility in women have circulated on social media [56]. Lastly, the politicization and polarization of news coverage surrounding the COVID-19 pandemic heavily influenced the public’s attitude to COVID-19 response policies [55,57-60]. Taken together, trouble with media outlets as trustworthy sources further supports the use of unbiased answer sources such as government agencies.

### Recommendations

Above all, we recommend that individuals consider health care professionals as the primary source of information regarding COVID-19 vaccines. However, in cases where access to a health care professional is limited, web-based sources unquestionably present opportunities to quickly provide high-quality and accurate information regarding COVID-19 vaccines. We agree with Mills and Sivelä [61] that a successful COVID-19 vaccination campaign depends on gaining the public’s trust in health care systems and government agencies, such as the Centers for Disease Control and Prevention and the World Health Organization, while also minimizing vaccine misinformation. Additionally, government sources must strive to translate scientifically dense literature into easily understandable information that answers widespread concerns. Therefore, the dissemination of this study’s findings may promote the public’s trust in these institutions as we have shown that government and academic sources provided the most transparent and highest-quality information addressing COVID-19 vaccine–related concerns.

Google recently demonstrated their willingness to support these COVID-19 vaccination campaigns by collaborating with Ohio State University to combat COVID-19 misinformation [62]. This partnership aims to ensure that people receive accurate information about COVID-19 vaccines to increase the public’s confidence and willingness to be vaccinated. Thus, in alignment with Google’s current intentions, we recommend that all COVID-19 vaccine FAQs be linked to government and academic answer sources; this would provide people with transparent and quality vaccine information. At a minimum, FAQs on safety and efficacy should be answered by government sources, as safety and efficacy concerns are among the primary drivers of COVID-19 vaccine hesitancy [39-42].

### Strength and Limitations

Our study’s primary strength is the incorporation of Google FAQs as a novel source of insight regarding millions of individual inquiries about COVID-19 vaccines, which is an application of methodology adapted from the published literature [21,26-28,34,35] and improved upon herein. Using FAQs generated by Google to explore the content of concerns regarding COVID-19 vaccine safety and efficacy may prevent common limitations of survey studies such as low response rates, reporting biases, and selection bias. Additionally, Google’s large data set is continuously analyzed in real time and may offer improved and more specific targets when approaching the public’s medical concerns. All classifications and assessments were performed in a masked duplicate fashion in accordance with standards set by the Cochrane Review and experts in the meta-research field [63,64] with high interrater reliability between investigators.

Our study is not without limitations though, such as those due to the dynamic nature of Google’s search outputs. As searching for COVID-19 vaccine–related information continues, new and updated FAQs will be generated, limiting the generalizability of our study to the time when our search was performed. Additionally, the transparency and quality assessments we used do not check for information accuracy, as this would require source-by-source comparison to generally accepted truths regarding COVID-19 vaccines, rendering our assessments as gauges of information transparency and not of information accuracy. Lastly, the categorizing of FAQs and answer sources was limited owing to their subjectivity. Although the categories were developed in line with previous reports and had high interobserver reliability, there is still potential for overlap between categories.

### Conclusions

The expedient development and approval of COVID-19 vaccines is the culmination of the world’s greatest scientific achievements; however, without positive public reception and adequate counseling and education, COVID-19 vaccination efforts may be hindered. Using Google allowed us to obtain a list of FAQs based on millions of searches for content related to COVID-19 vaccines, which reflected widespread and common concerns. We found that the most common COVID-19 vaccine–related questions pertained to vaccine safety and efficacy, which is supported by the findings of survey studies. We found that government and academic sources provided the most transparent and highest-quality web-based information for answering the public’s most frequently asked questions about COVID-19 vaccines. Recognizing common concerns about COVID-19 vaccines may better assist health care professionals, researchers, and government agencies in improving vaccination efforts. Ensuring a successful vaccination campaign requires the public’s trust, which may be enhanced through the availability of high-quality and transparent COVID-19 vaccine information, such as that provided by government sources.

## Abbreviations

FAQ: frequently asked question
JAMA: Journal of the American Medical Association
